# Evidence-Based Approach to Cerebral Vasospasm and Delayed Cerebral Ischemia: Milrinone as a Therapeutic Option—A Narrative Literature Review and Algorithm Treatment Proposition

**DOI:** 10.3390/neurolint17030032

**Published:** 2025-02-21

**Authors:** Pedro Batarda Sena, Marta Gonçalves, Bruno Maia, Margarida Fernandes, Luís Bento

**Affiliations:** 1 Intensive Care Department, Serviço de Saúde da Região Autónoma da Madeira, 9000-177 Funchal, Portugal; 2 Intensive Care Department, Unidade Local de Saúde de São José, Rua José António Serrano, 1150-199 Lisbon, Portugal

**Keywords:** aneurysmal subarachnoid hemorrhage, milrinone, neurocritical care, cerebral vasospasm, delayed cerebral ischemia

## Abstract

Aneurysmal subarachnoid hemorrhage (aSAH) is a severe neurocritical condition often complicated by cerebral vasospasm (CVS), leading to delayed cerebral ischemia (DCI) and significant morbidity and mortality. Despite advancements in management, therapeutic options with robust evidence remain limited. Milrinone, a phosphodiesterase type 3 (PDE3) inhibitor, has emerged as a potential therapeutic option. Intravenous milrinone demonstrated clinical and angiographic improvement in 67% of patients, reducing the need for mechanical angioplasty and the risk of functional disability at 6 months (mRS ≤ 2). Side effects, including hypotension, tachycardia, and electrolyte disturbances, were observed in 33% of patients, occasionally leading to early drug discontinuation. Based on the evidence, we propose a treatment algorithm for using milrinone to optimize outcomes and standardize its application in neurocritical care settings.

## 1. Introduction

Aneurysmal subarachnoid hemorrhage (aSAH) is a severe neurovascular condition resulting from the spontaneous rupture of an intracranial aneurysm, leading to subarachnoid blood accumulation and significant morbidity and mortality [1,2]. It affects 6 to 16 individuals per 100,000 annually, with notable regional disparities: higher incidence rates are reported in East Asia and parts of Europe [1,2]. At the same time, resource-limited settings face substantial diagnostic and therapeutic challenges [1,2]. Women are predominantly affected, with a peak incidence between 55 and 62 years of age [3,4,5]. Despite advancements in microsurgical and endovascular interventions, global mortality rates and severe disability remain persistently high, affecting approximately 30% of cases [4,6,7,8,9].

Complications such as cerebral vasospasm (CVS) and delayed cerebral ischemia (DCI) further exacerbate outcomes, with DCI—a significant determinant of poor prognosis—occurring in 25–30% of patients [4,10,11]. This condition emerges within a critical temporal window, providing preventive and therapeutic opportunities. Diagnostic approaches include computerized tomography (CT) and magnetic resonance imaging for detecting irreversible infarctions, alongside non-invasive methods like transcranial Doppler (TCD) and CT perfusion imaging to identify vasospasm’s impact on cerebral perfusion [12,13,14,15,16]. Nevertheless, effective treatment strategies remain limited and inconsistent across regions [15,16,17].

This manuscript adopts a narrative review approach due to the nuanced and multifaceted nature of its topic. Unlike systematic reviews, which adhere to rigorous inclusion criteria and predefined search methodologies, narrative reviews prioritize synthesizing diverse evidence to comprehensively understand a specific subject [18]. In this case, we summarize the clinical evidence of the therapeutic role of milrinone in cerebral vasospasm, which requires integrating mechanistic insights, clinical data, and expert interpretations. This format is particularly suitable given the complexity of the subject and the limited pool of high-quality studies directly addressing milrinone in this context. A narrative review also facilitates the exploration of clinical implications and the development of actionable treatment algorithms, which are central to the manuscript’s objectives [19]. This approach ensures a thorough and practical evaluation of the available data by allowing flexibility in the study selection and evidence integration. However, we will transparently acknowledge the inherent limitations of this format, including potential selection bias, in the limitations section.

Historically, CVS management relied on nonspecific and often suboptimal strategies, such as triple-H therapy, which posed significant risks. While mechanical angioplasty was effective, it was restricted to specialized centers with inherent procedural challenges and risks. Pharmacological advances, notably nimodipine and milrinone, represent a paradigm shift, offering targeted therapies with enhanced safety profiles [1,2,20,21,22].

Milrinone, in particular, has emerged as a pivotal agent in CVS management. Its dual mechanism of action—direct vasodilation via phosphodiesterase type 3 (PDE3) inhibition and inotropic support—addresses both vascular and perfusion deficits. By improving cerebrovascular autoregulation, reducing endothelial dysfunction, and mitigating neuroinflammation, milrinone offers comprehensive benefits in addressing the multifaceted pathophysiology of CVS [1,23,24,25,26]. However, its clinical application necessitates vigilant monitoring due to potential side effects, including hypotension and electrolyte imbalances [1,20,27].

This article evaluates the current evidence supporting milrinone’s role in CVS management. However, it is essential to emphasize that the proposed protocol and therapeutic orientations outlined in this review are based on the synthesis of available evidence. This aims to provide a logical framework to support clinicians in integrating milrinone into neurocritical care. These proposals should not be interpreted as definitive clinical guidelines but rather as a structured interpretation of existing data. Their application should be carefully adapted to established protocols, local resources, and multidisciplinary collaboration to ensure optimal patient care.

## 2. Literature Review

Aneurysmal subarachnoid hemorrhage (aSAH) is associated with multiple complications in both the acute and subacute phases, including CVS and DCI, which significantly contribute to morbidity and mortality. CVS, characterized by the narrowing of cerebral arteries, results from a complex interplay of endothelial dysfunction, neuroinflammation, and impaired autoregulatory mechanisms. These processes culminate in hypoperfusion and ischemic injury, often leading to long-term neurological deficits [2]. Delayed cerebral ischemia, affecting 20–40% of aSAH cases, is the predominant cause of poor functional outcomes in these patients [22,23].

Vasospasm typically manifests between the 3rd and 14th day after the hemorrhagic event, peaking around the 7th day [1,20,27,28,29]. Its diagnosis relies on cerebral angiography, considered the gold standard, and transcranial Doppler (TCD), which provides a noninvasive bedside assessment with approximately 90% sensitivity [20]. Clinically, vasospasm is characterized by worsening pre-existing deficits or the emergence of new neurological symptoms, often correlating with ischemic changes on imaging. Although CVS and DCI frequently coexist, they may occur independently [1,20,23].

Given the high prevalence and clinical impact of these complications, daily TCD monitoring is recommended, particularly in sedated patients with subtle clinical signs. Cerebral angiography is a diagnostic and therapeutic tool, enabling interventions such as angioplasty [27,30,31,32].

While nimodipine remains the only pharmacological agent with robust evidence for vasospasm prevention, its efficacy in treating established vasospasm is limited. Alternative approaches have shown promise, including intra-arterial nicardipine and nimodipine and systemic vasodilators like milrinone. Comparative analyses reveal that milrinone’s dual action—vasodilation and inotropic support—provides distinct advantages in improving cerebrovascular hemodynamics [27,31]. For example, intra-arterial nimodipine effectively reverses vasospasm but requires specialized equipment and expertise, limiting its widespread use in resource-constrained settings. Conversely, milrinone’s systemic administration offers broader applicability with comparable clinical outcomes [1,20,23,27].

Despite these advancements, existing studies exhibit significant methodological limitations, including small sample sizes, protocol heterogeneity, and a lack of randomized controlled trials. Additionally, regional disparities in the availability and application of milrinone underscore the need for standardized guidelines to ensure equitable access and optimized care [23,27,31].

Table 1 summarizes the key studies exploring milrinone’s role in CVS management:

## 3. Classification of Vasospasm

Vasospasm can be classified as mild (stenosis corresponding to <25%), moderate (25–50%), or severe (>50%). In the case of the anterior circulation, where we use the TCD, vasospasm is classified according to the mean blood flow velocities and the Lindegaard Index (LI—the ratio between the mean velocity in the intracranial artery and the corresponding mean velocity in the internal carotid artery that supplies it) [23,24,28]. The classification criteria are summarized in Table 2, which outlines the thresholds for mean flow velocities and Lindegaard Index values used to categorize the severity of vasospasm [23,24,28]:

While the TCD is a valuable bedside tool, it has limitations. Its accuracy depends on the operator’s expertise, and it may be less reliable in patients with poor acoustic windows or complex vascular anatomy. Additionally, TCD focuses on velocity changes, which do not always correlate with functional hemodynamic significance [20,29].

In posterior circulation, the index used is the Sviri Index, which is determined by the ratio between the mean flow velocities in the basilar/posterior cerebral arteries and the average velocity in both vertebral arteries [33]. Table 3 presents the classification criteria for vasospasm in posterior circulation based on mean flow velocities and the Sviri Index, categorizing it as possible, moderate, or severe.

The Sviri Index provides a structured approach to classifying vasospasm. However, baseline anatomic variations and coexisting pathologies can influence it in cases where TCD findings are equivocal; further imaging with CT or MR angiography is often required [33].

Cerebral angiography classifies CVS not based on circulation velocities but rather by identifying luminal filling defects and based on cerebral hemodynamics [33]. It remains the gold standard for diagnosing vasospasm in both anterior and posterior circulations. Unlike TCD-based indices, angiography allows direct visualization of luminal narrowing and hemodynamic alterations [34]. However, it is an invasive procedure with associated risks, including stroke and vessel injury. Despite these risks, angiography provides critical insights into the severity and distribution of vasospasm, informing therapeutic decisions such as chemical or mechanical angioplasty [27,35]:

- Chemical angioplasty involves the direct intra-arterial administration of vasoactive drugs to the cerebral circulation. This approach effectively targets localized vasospasm with fewer secondary systemic effects but requires specialized expertise [35].
- Mechanical angioplasty: Utilizes inflatable balloons or stents to dilate affected arterial segments. It provides immediate relief but is associated with procedural risks and limited availability [35].

Intravenous milrinone has emerged as a promising therapy for treating established vasospasm, effectively addressing vascular and perfusion deficits. Studies highlight its potential to reduce delayed cerebral ischemia (DCI) and improve functional outcomes, although further research is needed to standardize its use [27,35].

## 4. Milrinone Pharmacodynamics

Milrinone, a selective PDE3 inhibitor, has established itself as a cornerstone in the management of cerebral vasospasm (CVS) associated with aneurysmal subarachnoid hemorrhage (aSAH). It is a PDE3 inhibitor that has a positive inotropic effect on the myocyte and a vasodilatory impact on the smooth muscle of the vessels. It has a half-life of about 2.5 h and a distribution volume of 0.38 L/kg. It is a highly eliminated drug that is 70% bound to plasma proteins [1].

Its pharmacodynamic profile encompasses a multifaceted approach, including vascular smooth muscle relaxation, enhanced myocardial contractility, and anti-inflammatory effects, making it a versatile agent in neurocritical care [1,23]. By inhibiting PDE3, an enzyme responsible for the degradation of cyclic adenosine monophosphate (cAMP), milrinone increases intracellular cAMP levels in vascular smooth muscle cells and cardiac myocytes. This elevation in cAMP promotes the relaxation of vascular smooth muscle by reducing intracellular calcium concentrations, effectively decreasing vascular tone and enhancing cerebral blood flow. This vasodilatory effect, mediated through elevated cAMP, is pivotal in counteracting pathological vasoconstriction, enabling cerebral vessels to dynamically adapt to systemic pressure changes and restore perfusion in ischemic territories [1,20,23,27].

In cardiac myocytes, increased cAMP enhances calcium influx during systole, thereby improving myocardial contractility and overall cardiac output. This dual mechanism ensures adequate systemic and cerebral perfusion, particularly in hemodynamically unstable patients at high risk of delayed cerebral ischemia (DCI). Furthermore, milrinone’s vasodilatory action extends to the endothelium, enhancing nitric oxide bioavailability and reducing oxidative stress, mitigating endothelial dysfunction, a critical factor in the pathophysiology of CVS [24,25,26,29]. Emerging evidence also suggests that milrinone possesses significant anti-inflammatory properties, including the modulation of inflammatory cytokines and reduction in leukocyte-endothelial interactions. These effects contribute to preserving cerebrovascular endothelial integrity and attenuate the neuroinflammatory cascade, essential for maintaining autoregulatory responses and preventing further ischemic damage [24,25,26,29].

The clinical implications of milrinone’s pharmacodynamics are profound. Studies have demonstrated significant improvements in cerebral blood flow following its administration, as evidenced by reductions in mean flow velocities measured via transcranial Doppler (TCD) [2]. Additionally, its dual action on vascular tone and cardiac function addresses the vascular and perfusion deficits that are hallmarks of CVS. This comprehensive approach has been associated with improved functional outcomes, including a reduced incidence of DCI and better modified Rankin Scale (mRS) scores at six months [33,34]. The Montreal Protocol, a widely referenced standard for milrinone administration, reported angiographic improvement in 67% of patients, with a complete resolution of vasospasm in 30%. These findings underscore the potential of milrinone to enhance recovery and optimize long-term outcomes [23,36].

Milrinone’s pharmacodynamic effects are dose-dependent, allowing for tailored treatment strategies. Low-to-moderate doses (0.75–1.25 µg/kg/min) primarily achieve vasodilation with minimal systemic effects, making them suitable for patients with mild-to-moderate vasospasm. Higher doses (1.5–2.0 µg/kg/min) provide additional inotropic support, which is particularly beneficial for patients with concurrent cardiac dysfunction. The maximum recommended dose of 2.5 µg/kg/min is reserved for select cases under close hemodynamic monitoring to balance therapeutic benefits against the risk of adverse effects [23,25,37].

While milrinone offers significant therapeutic advantages, its use is not without challenges. Excessive vasodilation can lead to systemic hypotension, compromising cerebral perfusion. This complication often necessitates volume resuscitation and the administration of vasopressors, such as norepinephrine, to maintain adequate cerebral perfusion pressure [23,29]. Tachycardia, another potential side effect, may require dose adjustments, especially in patients with limited cardiac reserve [26]. cAMP levels can affect renal function and electrolyte balance, especially potassium levels, requiring proactive monitoring to prevent arrhythmias [24]. Therefore, continuous monitoring of hemodynamic parameters and promptly correcting electrolyte imbalances are vital to minimizing these risks and ensuring safe and effective treatment.

Emerging research has begun to explore the impact of milrinone on specific inflammatory biomarkers, such as interleukin-6 (IL-6) and tumor necrosis factor-alpha (TNF-α), which are implicated in the pathogenesis of neuroinflammation in CVS. Preliminary findings suggest that milrinone may attenuate the release of these cytokines, further supporting its role in modulating the inflammatory response [36,38,39,40]. This aspect of its pharmacodynamics highlights its potential as part of a comprehensive therapeutic strategy that extends beyond vasodilation and inotropic support.

Milrinone’s versatility is particularly valuable when first-line therapies fail or are insufficient. However, its clinical application in resource-limited settings poses additional challenges. The need for advanced monitoring equipment and multidisciplinary expertise can limit its accessibility. Strategies to address these barriers include developing simplified protocols and training programs for healthcare providers to optimize its use in diverse clinical environments. Collaborative research efforts focusing on cost-effectiveness and integrating milrinone into existing treatment frameworks are crucial to expanding its applicability [23,28].

In conclusion, milrinone’s pharmacodynamic properties underscore its potential as a cornerstone therapy for managing CVS. By addressing both vascular spasms and perfusion deficits, it bridges critical gaps in the treatment of aSAH-associated complications. Ongoing research into its long-term effects, impact on inflammatory pathways, and integration with adjunctive therapies will further refine its role, paving the way for enhanced patient care and improved neurocritical outcomes [26,28,37].

## 5. Treatment Orientations

Milrinone therapy is most beneficial in patients with moderate-to-severe CVS who have failed to respond to first-line treatments. Key criteria include the following [1,23,26,29]:

1. Documented moderate-to-severe vasospasm on TCD or angiography [20,27].
2. Clinical signs consistent with DCI, such as the following:
  ○ Focal neurological deficits (e.g., hemiparesis and aphasia) [1,23].
  ○ A reduced level of consciousness sustained over 1 h [10,22].
3. The exclusion of alternative etiologies for neurological deterioration, such as hydrocephalus, metabolic imbalances, or seizures [4,35].

Treatment with milrinone is not recommended in certain conditions because of its pharmacodynamic effects on the cardiovascular system.

In Table 4, we enumerate the Mais absolute and relative contraindications.

Absolute contraindications include hypersensitivity to milrinone [23], as allergic reactions can lead to severe hemodynamic instability. Patients with severe aortic stenosis or obstructive hypertrophic cardiomyopathy should also be excluded from therapy [24] since milrinone’s vasodilatory and inotropic effects may worsen left ventricular outflow tract obstruction, resulting in further hemodynamic deterioration. Additionally, refractory hypotension is a strict contraindication [28], as the vasodilatory properties of milrinone can further compromise cerebral and systemic perfusion in patients with an already unstable hemodynamic profile. Finally, significant renal impairment (GFR < 30 mL/min) poses a risk of drug accumulation since milrinone is primarily excreted through the kidneys, necessitating dose adjustments or alternative therapies for these patients [31].

Relative contraindications require careful clinical assessment before starting therapy. Cardiac dysfunction, especially in patients with elevated troponin levels or reduced ejection fraction, may limit the benefits of milrinone since its positive inotropic effects could increase myocardial oxygen demand and worsen myocardial injury [25]. Likewise, severe electrolyte imbalances, such as hypokalemia and hypermagnesemia, raise concerns due to milrinone’s potential to cause arrhythmias [26].

## 6. Selection of Patients for Milrinone Therapy

Milrinone therapy is recommended for patients with excluded aneurysms who satisfy specific clinical and imaging criteria, ensuring focused treatment for cerebral vasospasm (CVS) and delayed cerebral ischemia (DCI) [23,24,28,38,42].

In the Table 5, we resume and describe the eligibility criteria for milrinone therapy.

A diagnosis of delayed cerebral ischemia (DCI) is considered when a patient presents with a new focal neurological deficit lasting at least one hour, which cannot be attributed to other clinical conditions such as hydrocephalus, metabolic imbalances, or seizures [25,36]. In Table 6, we summarize and detail the criteria for neurological deficits or symptoms that suggest neurological deterioration.

## 7. Initial Assessment

A comprehensive neurological examination is required to assess aneurysmal subarachnoid hemorrhage (aSAH), incorporating the FOUR score to evaluate consciousness and brainstem function [41,42]. This examination should be performed in all patients to identify potential neurological deterioration and assess for cerebral vasospasm (CVS) when suspected [23,24,28].

Hemodynamic monitoring is essential for guiding treatment decisions. Continuous invasive blood pressure measurement, heart rate assessment, and ECG monitoring pro-vide critical data on perfusion and cardiovascular function [28,36]. In selected cases, transthoracic echocardiography (TTE) assists in evaluating cardiac function, particularly before and after therapeutic interventions [28,30,36]. For patients with hemodynamic instability or requiring high-dose vasopressor therapy, advanced hemodynamic monitoring may be considered to optimize circulatory support [28,36,42,48].

Laboratory evaluation supports clinical decision-making and should include base-line and daily monitoring of complete blood count [28,36,42], electrolyte levels (Na⁺, K⁺, P⁺, Mg^2^⁺, and Ca⁺) [28,36,42,49], and tests for renal and liver function [28,36,42,49,50]. In cases where myocardial dysfunction is suspected, cardiac enzyme analysis may provide additional diagnostic insight and influence management strategies [49].

## 8. Initiation of Milrinone Infusion

The initiation of milrinone therapy should be guided by transcranial Doppler (TCD) and cerebral angiography findings to confirm the severity of cerebral vasospasm (CVS) [24,28,29]. Based on existing evidence, the administration protocol is organized into stepwise interventions, ensuring optimal dose titration and patient safety.

Milrinone therapy is initiated using a structured dose titration approach, which ensures appropriate administration based on the severity of the vasospasm and the patient’s response.

Treatment begins with an initial bolus of 0.1 mg/kg administered over 10 min to achieve therapeutic plasma levels [35]. Following this, a continuous infusion at 0.75 µg/kg/min is initiated, providing sustained vasodilatory and inotropic effects [24].

**Figure 1:**
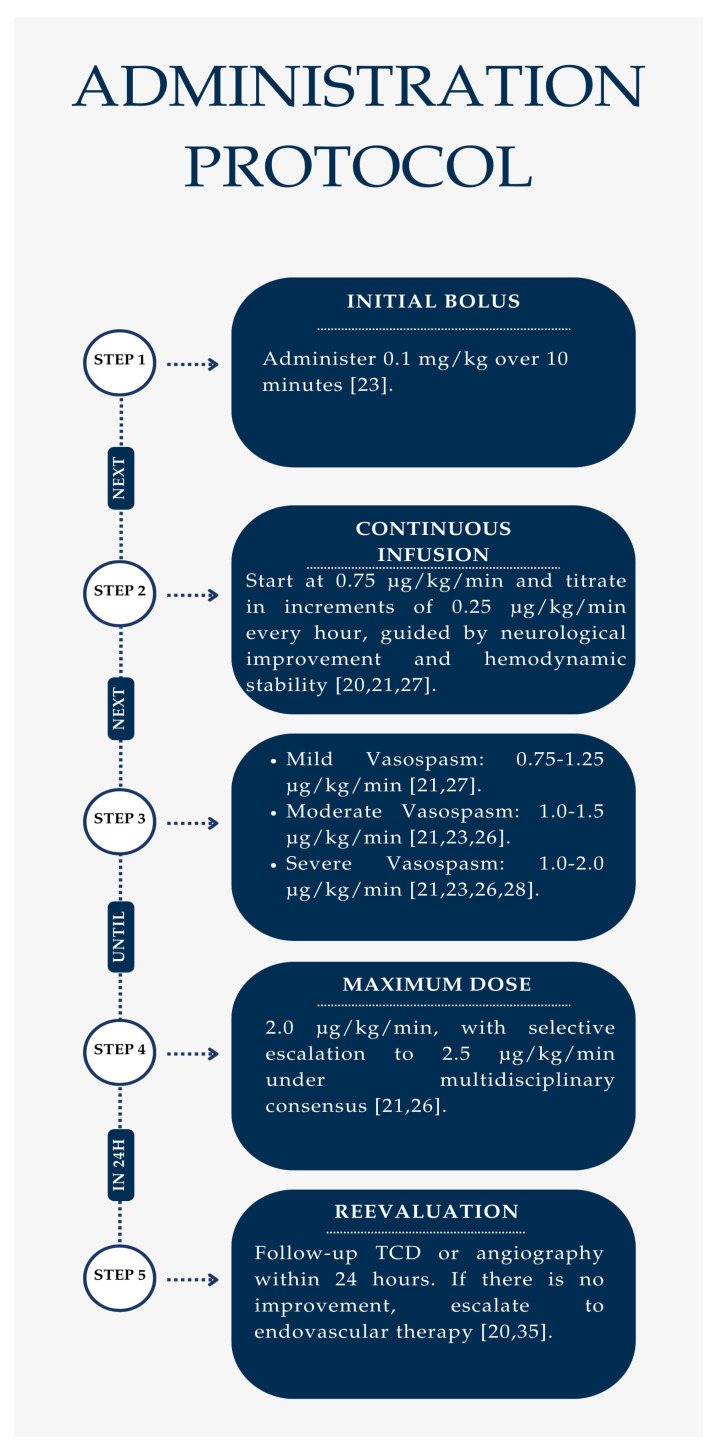
Milrinone dose and titration flowchart summary [20,21,23,26,27,28,35].

The infusion rate should be titrated hourly in increments of 0.25 µg/kg/min, adjusting the dose based on neurological improvement and hemodynamic stability. The appropriate dosage depends on the severity of cerebral vasospasm (CVS), as shown in flowchart 1 (Figure 1).

The maximum recommended dose is 2.0 µg/kg/min, which balances therapeutic efficacy with the risk of hypotension and systemic vasodilation. The literature supports dose escalation up to 2.5 µg/kg/min in selected cases, particularly under multidisciplinary review and continuous hemodynamic monitoring [24,28,51].

Continuous monitoring and intervention adjustments are required during milrinone therapy to optimize cerebral perfusion and hemodynamic stability.

Vasopressor support is crucial when the mean arterial pressure (MAP) cannot be maintained at ≥90 mmHg. In such cases, norepinephrine (NA) may be administered at doses up to 0.5 µg/kg/min. If MAP remains insufficient, vasopressin should be considered as an additional agent to further enhance perfusion pressure [29,48,52,53]. 

Advanced hemodynamic monitoring is recommended for patients receiving milrinone doses greater than 1.0 µg/kg/min while requiring norepinephrine at doses exceeding 0.5 µg/kg/min. In such cases, transpulmonary thermodilution should be employed to evaluate cardiac output, preload status, and systemic vascular resistance, ensuring an individualized and targeted approach to hemodynamic management [29,48,52,53].

**Figure 2:**
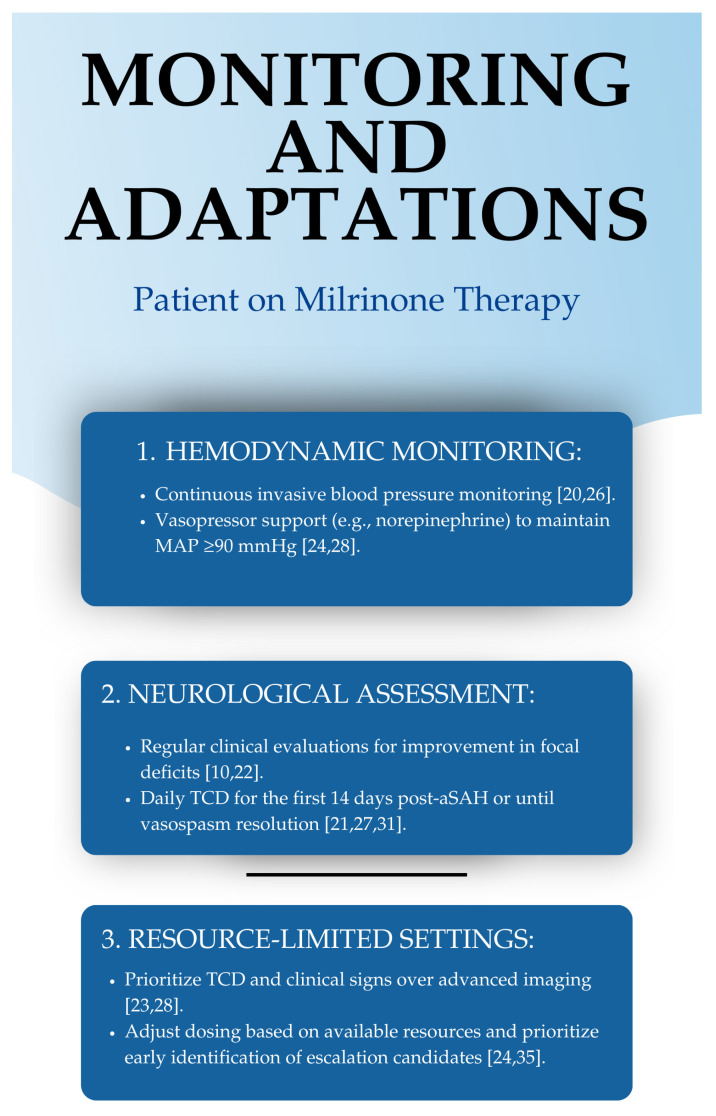
Hemodynamic and neurological monitoring orientations [10,20,21,22,23,24,26,27,28,31,35].

In cases where clinical deterioration persists despite treatment, it is crucial to reassess cerebral perfusion. If there is no neurological improvement or signs of worsening cerebral ischemia, repeat cerebral angiography should be conducted, and endovascular treatment should be considered to restore adequate cerebral blood flow and minimize secondary is-chemic injury [35]. These guidelines are illustrated in Figure 2.

## 9. Monitoring of Milrinone Treatment

The administration of milrinone requires structured monitoring to ensure therapeutic efficacy while minimizing complications, particularly systemic hypotension due to its vasodilatory effects. A comprehensive approach incorporating clinical assessment, neurological monitoring, and hemodynamic stabilization is essential to optimize patient outcomes [24,26,36].

Neurological assessment should be performed every 30–60 min until clinical improvement is observed or the maximum therapeutic dose is reached. Once the patient stabilizes, evaluations should continue at 8-h intervals to monitor for delayed neurological deterioration [36]. In addition to clinical examinations, daily transcranial Doppler (TCD) evaluations should be conducted for at least 14 days post-event, especially in patients who have undergone aneurysm exclusion [28]. If vasospasm persists, TCD monitoring should be extended until resolution is confirmed, ensuring timely intervention if deterioration occurs [21,51].

For patients who are sedated or have altered consciousness, routine clinical assessment may be insufficient to detect cerebral perfusion deficits. In these cases, multimodal neuromonitoring techniques should be employed, including EEG with spectral analysis, brain tissue oxygenation (PtiO_2_) monitoring, cerebral microdialysis, or near-infrared spectroscopy (NIRS). These tools enable the early detection of ischemic injury, allowing for adjustments in therapy before irreversible damage occurs [2,42,43,44,45,46,47].

Given the vasodilatory effects of milrinone, maintaining hemodynamic stability is critical to preventing secondary cerebral ischemia. If hypotension develops, the first step is to withdraw all hypotensive agents, except nimodipine, to prevent further reductions in blood pressure while preserving its neuroprotective properties [26,37]. When volume deficits are suspected, volume resuscitation with crystalloids should be initiated to restore euvolemia, ensuring adequate circulating volume before escalating vasopressor support [24,26,37]. If further intervention is required, norepinephrine infusion should be initiated, targeting a mean arterial pressure (MAP) of at least 90 mmHg or optimizing cerebral perfusion pressure (CPP) to prevent secondary ischemic injury [28].

For patients requiring high-dose vasopressor support, advanced hemodynamic monitoring is recommended. When norepinephrine doses exceed 0.5 µg/kg/min and milrinone infusion surpasses 1.0 µg/kg/min, transpulmonary thermodilution should be employed to assess cardiac output, preload status, and systemic vascular resistance, ensuring precise hemodynamic optimization [36,48,52]. If hemodynamic instability persists despite these interventions, milrinone infusion should be gradually reduced by 0.25 µg/kg/min every 1–2 h, striking a balance between therapeutic vasodilation and the risk of excessive hypotension [26,36].

Imaging: All patients should undergo a CT-CE study within the first 48 h of treatment initiation [21,24,29,34].

## 10. Other Care (For All Patients)

Patients undergoing treatment for aneurysmal subarachnoid hemorrhage (aSAH) who receive milrinone therapy need comprehensive supportive care to optimize outcomes and reduce secondary complications. Administering nimodipine at a dose of 60 mg every 4 h for 21 days remains the standard pharmacological approach for preventing delayed cerebral ischemia (DCI) [23,36]. Maintaining euvolemia is crucial to support cerebral perfusion, requiring balanced fluid management and regular assessment of renal function to avoid fluid overload or hypovolemia [51].

Systemic homeostasis plays a crucial role in neurological recovery, requiring strict control of body temperature and glucose levels to minimize secondary metabolic stress on the injured brain [21,51]. A hyperosmolar environment, characterized by a serum sodium concentration exceeding 140 mEq/L, is often preferred to help regulate intracranial pressure (ICP) and sustain cerebral perfusion [21,51]. Furthermore, diligent monitoring and correction of electrolyte imbalances and acid-base disturbances are necessary to avert secondary cardiovascular and neurological complications [21,51]. Hematological optimization should also be taken into account, with transfusion support aimed at keeping hemoglobin levels above 9.0 g/dL when clinically warranted, ensuring sufficient oxygen delivery to ischemic brain tissue [54,55,56].

## 11. Discontinuation

Milrinone therapy should continue until vasospasm resolution is confirmed through serial transcranial Doppler (TCD) assessments or cerebral angiography [23,24,26]. To minimize withdrawal-related hemodynamic instability, dose tapering should be performed gradually, reducing the infusion rate by 0.25 µg/kg/min every 12–24 h until it reaches 0.75 µg/kg/min, at which point therapy can be discontinued [26].

If clinical or imaging deterioration occurs during tapering, a bolus dose of 0.05 mg/kg may be given, followed by an increase in the infusion rate to the previously effective dose until hemodynamic stability is restored [24,26]. Therapy should be permanently stopped if severe side effects develop or if no clinical improvement is seen after 72 h of treatment at the optimal dose [18].

By implementing these supportive measures and ensuring a structured discontinuation process, the risks associated with milrinone therapy can be effectively managed while optimizing neurological recovery in patients with aSAH-related vasospasm.

## 12. Documentation and Communication

We suggest documenting all clinical and imaging evaluations, dosage adjustments, and patient clinical responses in a standardized table (Table 7).

Team communication: maintain regular communication with the multidisciplinary team, including neurosurgery and neuroradiology.

## 13. Protocol Schematics

**Figure 3:**
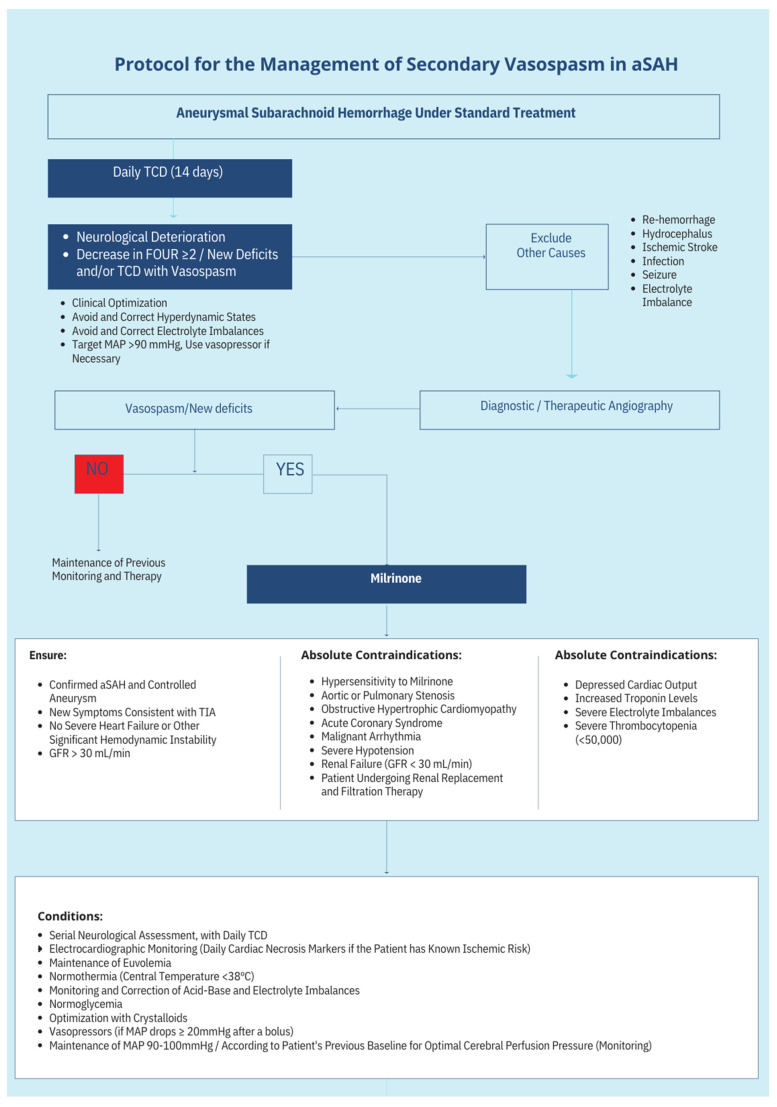
Proposed algorithm for the management of CVS in aSAH using Milrinone. The algorithm outlines a step-by-step approach to diagnosing and treating CVS in patients with aSAH. It includes criteria for patient selection, initiation of milrinone therapy, dosage titration, and indications for advanced monitoring and endovascular treatment.

**Figure 4:**
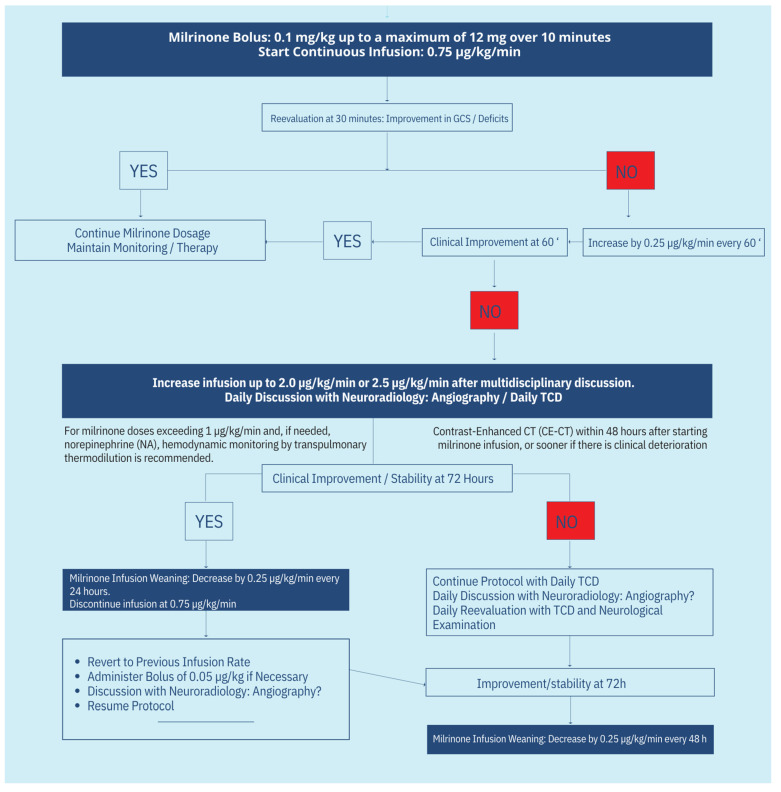
Protocol for milrinone administration in CVS. This figure continues the protocol with a detailed protocol for milrinone administration, including initial bolus dosing, continuous infusion titration based on the severity of vasospasm, and steps for managing drug-related complications such as hypotension. [1,23,24,26,36,48,51].

The treatment protocol’s complete schematics can be seen in Figure 3 and Figure 4.

## 14. Discussion

The available evidence highlights the effectiveness of milrinone as an adjunctive therapy for CVS management in patients with aSAH. Its incorporation into neurocritical care signifies a change in managing CVS and DCI, providing an alternative to conventional therapies, which have limitations. Nimodipine remains the standard for vasospasm prevention, but its effectiveness in treating established vasospasm is restricted. Milrinone offers a targeted alternative, combining vasodilation and inotropic support, resulting in improved cerebrovascular perfusion and reduction in ischemic injury. Standardized protocols, such as those outlined in the Montreal Protocol and MILRISPAN study, have facilitated the structured implementation of milrinone, improving reproducibility and clinical outcomes [24,30].

Comparative studies highlight both the advantages and limitations of existing therapeutic approaches. Intra-arterial nicardipine effectively reverses vasospasm, but its use is limited by the need for specialized expertise and infrastructure, making it less feasible in resource-constrained environments. Systemic milrinone, in contrast, offers a more accessible alternative, demonstrating both angiographic and clinical efficacy [26,51]. Another widely utilized option, mechanical angioplasty, provides immediate relief but is limited to refractory cases due to procedural risks [20,42].

Despite its advantages, milrinone’s clinical application presents challenges. Continuous hemodynamic monitoring requires advanced critical care resources, which are not always available. Additionally, side effects such as hypotension and electrolyte imbalances require vigilant monitoring and a multidisciplinary approach to treatment [26,28]. These limitations highlight the necessity of tailoring treatment protocols to individual patient needs and available institutional resources.

Future Directions

Further research is needed to refine milrinone’s role in neurocritical care, with a particular focus on the following:

- Large-scale randomized controlled trials (RCTs) to validate its efficacy and safety.
- Biomarker-based patient stratification to predict treatment response and optimize therapy.
- Combination therapies exploring synergies with other pharmacologic agents or interventions.
- The integration of advanced monitoring technologies, such as automated TCD and perfusion MRI, to refine patient selection and treatment adaptation.
- Standardized protocols, such as the Montreal Protocol, have demonstrated the reproducibility of milrinone administration, particularly in reducing the incidence of DCI and improving long-term neurological outcomes. However, variability in patient response, hemodynamic instability, and resource limitations necessitate a personalized and monitored approach.

Clinical Recommendations

To ensure the safe and effective use of milrinone, clinical implementation should follow evidence-based principles, including the following:

- Protocol adherence to structured administration and monitoring strategies.
- A multidisciplinary approach, engaging neurosurgery, neurocritical care, and interventional neuroradiology teams.
- Careful patient selection, prioritizing those with moderate-to-severe vasospasm refractory to first-line therapies.
- Comprehensive hemodynamic and neurological monitoring to mitigate risks.
- Active participation in research and multicenter trials to further refine best practices for milrinone therapy.

While this review was conducted independently and without financial sponsorship, it is important to acknowledge potential limitations. As a narrative review, the study selection process is inherently subject to selection bias. Additionally, the heterogeneity of the available studies and the limited number of high-quality trials on milrinone for CVS impact the generalizability of findings. Differences in study design and patient populations also present challenges in translating evidence into routine clinical practice.

## 15. Conclusions

Milrinone has emerged as a promising therapeutic option for managing CVS and DCI in aSAH patients, offering a dual mechanism of vasodilation and inotropic support that differentiates it from conventional therapies. The Montreal Protocol and MILRISPAN study have contributed to its structured implementation, demonstrating improved functional outcomes and ischemic injury prevention.

Despite these benefits, careful patient selection and monitoring remain essential to mitigate risks, including hypotension and electrolyte disturbances. A multidisciplinary approach, integrating intensivists, neurologists, and interventional radiologists, is critical to optimizing therapeutic outcomes.

Further research should focus on large-scale RCTs, biomarker-driven therapy refinement, and cost-effectiveness analyses, particularly in resource-limited settings where treatment accessibility remains challenging. Expanding international collaboration and standardizing protocols will be crucial to maximizing milrinone’s role in neurocritical care.

By continuing to advance research and refine treatment protocols, milrinone can fill a critical gap in CVS management and improve the outcomes of patients with aSAH.

## Figures and Tables

**Table 1:** Literature summary—This table provides an overview of key studies examining the use of milrinone in the treatment of CVS. It highlights the study designs, sample sizes, primary findings, and associated limitations.

Study	Design	Sample Size	Key Findings	Limitations
Montreal Protocol (2012) [23]	Observational Cohort	45	Angiographic improvement in 67% of patients; functional improvement in 30%	Small sample size; observational design
MILRISPASM (2021) [26]	Controlled Before–After	120	Reduction in DCI; fewer mechanical interventions required	No randomized control
Bernier et al. (2021) [24]	Systematic Review	500+	Demonstrated safety and moderate efficacy of milrinone across multiple studies	Data heterogeneity
Comparative Study: Nimodipine [25]	Retrospective Cohort	80	Nimodipine superior for prevention; milrinone superior for treatment	Limited comparative data

**Table 2:** Classification of vasospasm.

Classification of Vasospasm	Mean Velocity	LI
Mild	<120 cm/s	3–4
Moderate	120–180 cm/s	5–6
Severe	>180 cm/s	>6

**Table 3:** Sviri Index.

Posterior Circulation	Mean Velocity	Sviri Index
Possible	70–85 cm/s	2–2.5
Moderate	>85 cm/s	2.5–3
Severe	>85 cm/s	>3

**Table 4:** Absolute and relative contraindications.

Category	Condition
Absolute	Hypersensitivity to milrinone [23].
	Aortic or pulmonary valve stenosis [24].
	Obstructive hypertrophic cardiomyopathy [26].
	Acute coronary syndrome or malignant arrhythmias [28].
	Severe refractory hypotension [41].
	Significant renal insufficiency (GFR < 30) and/or dialysis dependency [23,24,26,28,41].
Relative	Worsening cardiac output and elevated troponin levels, indicating underlying myocardial dysfunction [25].
	Severe electrolyte imbalances, including hypokalemia, hypermagnesemia, or other ionic disturbances that may exacerbate arrhythmias [36].

**Table 5:** Eligibility criteria for milrinone therapy.

Eligibility Criteria	Description
Clinical signs with or without vasospasm	Moderate-to-severe vasospasm documented on TCD or cerebral angiography [23,24,28].
No clinical changes but vasospasm detected	Vasospasm observed during daily TCD monitoring, confirmed by angiography [38,42].
No clinical changes but vasospasm suspected by alternative monitoring methods	PtiO_2_, NIRS, or EEG findings suggesting vasospasm, confirmed by angiography [2,43,44,45,46,47].

**Table 6:** New neurological deficit/symptom criteria.

Criterion	Description
Altered level of consciousness and/or orientation	Confirmed in at least two consecutive neurological evaluations [23,24,28,41].
Decline in FOUR score	Reduction in two or more points on the Full Outline of UnResponsiveness (FOUR) score [23,24,28,41].
Cranial nerve paresis or speech/language alteration	Includes apraxia, hemianopsia, neglect, and/or new focal motor deficit [23,24,28,41].
Rapid-onset neurological deficit	Symptoms appearing within a 4-h window [23,24,28,41].
Headache refractory to analgesia	Persistent headache despite appropriate analgesic treatment [23,24,28,41].

**Table 7:** Suggestion for data collection sheet.

Field	Description	Format
Patient ID	Unique identifier of the patient (anonymous).	Text/Numeric
Inclusion Date	Date of initiation of treatment with milrinone.	Date (DD/MM/YYYY)
Sex	Patient’s sex.	M/F
Age	Patient’s age at the time of treatment.	Numeric
Body Weight	Weight in kilograms for dosage calculations.	Numeric (kg)
Initial Diagnosis	Diagnosis related to vasospasm (e.g., aneurysmal SAH).	Text
Initial Vasospasm Severity	Classification (mild, moderate, or severe) based on TCD/angiography.	Text
Initial TCD Mean Velocity	Mean velocity in cm/s before starting treatment.	Numeric (cm/s)
Initial Lindegaard Index	Index calculated before initiating treatment.	Numeric
Initial Milrinone Dose	Initial dose administered.	Numeric (µg/kg/min)
Maximum Milrinone Dose	Maximum dose reached during treatment.	Numeric (µg/kg/min)
Treatment Duration	Total duration of milrinone administration.	Hours/Days
Initial Hemodynamics	Data such as MAP, HR, and other parameters before treatment.	Text
Adverse Effects	Record of adverse events (e.g., hypotension, tachycardia, etc.).	Text
Additional Interventions	Need for other treatments (e.g., angioplasty or other drugs).	Text
Clinical Improvement	Observation of clinical or neurological improvement.	Yes/No
Follow-Up Imaging (TCD/CT)	Results of tests performed during and after treatment.	Text
Associated Complications	Record of complications during or after treatment.	Text
Neurological Outcome (mRS)	Modified Rankin Scale score after 6 months.	Numeric (0–6)
General Comments	Additional relevant observations.	Text

## Data Availability

No new data were created or analyzed in this study.
